# Exosomal tetraspanins mediate cancer metastasis by altering host microenvironment

**DOI:** 10.18632/oncotarget.19119

**Published:** 2017-07-10

**Authors:** Jun Lu, Jun Li, Shuo Liu, Teng Wang, Alessandro Ianni, Eva Bober, Thomas Braun, Rong Xiang, Shijing Yue

**Affiliations:** ^1^ School of Medicine, Nankai University, Tianjin, China; ^2^ The State International Science & Technology Cooperation Base of Tumor Immunology and Biological Vaccines, Nankai University, Tianjin, China; ^3^ Department of General Surgery, Hefei Second People’s Hospital, Hefei, China; ^4^ Department of Cardiac Development and Remodeling, Max-Planck-Institute for Heart and Lung Research, Bad Nauheim, Germany

**Keywords:** tumor, cancer metastasis, tetraspanin, exosomes, cancer microenvironment

## Abstract

The metastases of malignant tumors develop through a cascade of events. The establishment of a pre-metastatic micro-environment is initiated by communication between tumors and host. Exosomes come into focus as the most potent intercellular communicators playing a pivotal role in this process. Cancer cells release exosomes into the extracellular environment prior to metastasis. Tetraspanin is a type of 4 times transmembrane proteins. It may be involved in cell motility, adhesion, morphogenesis, as well as cell and vesicular membrane fusion. The exosomal tetraspanin network is a molecular scaffold connecting various proteins for signaling transduction. The complex of tetraspanin-integrin determines the recruiting cancer exosomes to pre-metastatic sites. Tetraspanin is a key element for the target cell selection of exosomes uptake that may lead to the reprogramming of target cells. Reprogrammed target cells assist pre-metastatic niche formation. Previous reviews have described the biogenesis, secretion and intercellular interaction of exosomes in various tumors. However, there is a lack of reviews on the topic of exosomal tetraspanin in the context of cancer. In this review, we will describe the main characteristics of exosomal tetraspanin in cancer cells. We will also discuss how the cancer exosomal tetraspanin alters extracellular environment and regulates cancer metastasis.

## INTRODUCTION

In tumor tissues, diverse types of non-cancer cells intermingle with heterogeneous cancer cells [1]. Non-malignant cells and extracellular matrix (ECM) of the tumor are defined as the tumor stroma. Tumor progress is dependent on interactions within this complex micro-environment [2–4]. During the initiation, tumor growth beyond the threshold size of a few mm^3^ is largely determined by the regulation of tumor cell, non-tumor cell, angiogenesis, immunological system [5, 6]. Cancer micro-environment is a complex system of many elements including the extracellular matrix, growth factors, angi-ogenic factors, stromal cells and inflammatory cells. Initiating the formation of new blood vessels is important for tumor growth and survival [7, 8]. Bone-marrow-derived cancer promoting cells (including haematopoietic progenitor cells and macrophages) express efficiently tumor growth factors. Bone-marrow-derived cancer promoting cells house tumor-specific pre-metastatic sites to prepare cancer pre-metastatic niche formation and metastasis [9–11].

Exosomes are derived from multi-vesicular bodies (MVBs) and contain mRNA, miRNA, as well as enriched tetraspanins and tetraspanin-associated proteins [12, 13]. Tetraspanins are a major component of exosomes. It is likely that tetraspanins impact the functional activity of exosomes [14]. From current knowledge, there is strong evidence that tumor-derived exosomes play a major role in the interaction between tumor and host. The biogenesis and main physical characteristics have been defined with special focus on their oncogenic role [15–19]. The most important part of exosome biogenesis is the formation of intra-lumenal vesicles (ILVs) of MVBs. The endosomal sorting complex for transport (ESCRT-0, -I, II, -III) is a key component for exosome biogenesis. Numerous accessory proteins (including syndecan heparan) and syntenin, their cytoplasmic adaptor, control the formation of exosomes. ALIX, the interactive partner of syntenin, is required for exosome biogenesis [20–22].

The uptake of exosomes is mediated by the interaction between exosomal ligands and receptors of recipient cells. It is likely that there are several different mechanisms for the uptake of exosomes by target cells. Exosomes deliver content via internalization or by fusing the plasma membrane of the recipient cells [23–27]. Uptake of exosomes by target cells may accompany the transferring of mRNAs, miRNAs and proteins. The molecular activities dependent on exosomes uptake lead to reprogramming of the recipient cells [18, 28–30]. The published data has demonstrated that exosomal tetraspanins are involved in the mechanism of target cells uptake [31]. Besides carrying information into target cells, exosomes are also involved in remodelling the tumor micro-environment. Tumor exosomes confer new activities to stromal cells, endothelial cells, epithelial cells, fibroblasts and macrophages [32–34]. Exosomes-educated non-cancer cells promote tumor vascular permeability, immune escaping, angiogenic switch and highly drug resistant properties [35–41]. As signaling scaffolds, exosomal tetraspanins can induce a series of activations of target cells. Extracellular matrix is an important section of tumor micro-environment. Tumor exosomes remodeling extracellular matrix can prepare a path for cancer metastasis. Recently the conception, biogenesis, composition, secretion and cancer progress regulation of exosomes have been reviewed extensively [20–22, 35, 42]. The present review highlights tetraspanins that are involved in assembly of exosomes, exosomes uptake and exosomal tetraspanins regulating the alteration of cancer micro-environment.

## EXOSOMES: NEW INTERCELLULAR COMMUNICATORS

### Discovery and original biogenesis of exosomes

Exosomes was first discovered *in vitro* culture of sheep reticulocytes by Johnstone *et al.* in 1987 [43]. A decade later, a few investigations demonstrated that exosomes may be signaling carriers with proteins and bioactive molecules [44]. Additional studies showed further evidence that exosomes played the role of a communicator between cells [44–46]. Extracellular vesicles represent the large family of non-classical secretory vesicles, with microvesicles and exosomes being two sub-sets of this family.Exosomes—small extracellular vesicles of 30-100nm [42]—are secreted by multiple cells and distributed in all body fluids including blood, milk and urine. Exosomes are derived from the fusion of intraluminal vesicles of MVBs with the plasma membrane [47, 48]. The molecular composition of exosomes indicates their origin from intraluminal vesicles [49, 50]. Besides a set of common membrane and cytosolic molecules, the components of exosomes contain tetraspanins including CD9, CD37, CD53, CD63, CD81, CD82, CD151, and Tspan8 (CO-029/D6.1A). Exosomes harbour sub-sets of proteins contain integrins, ICAMs, MHC; vesicle transport associated molecules; cytoskeletal proteins; heat shock proteins (HSP); enzymes; signaling molecules, and so on [42, 51]. ESCRT complexes (ESCRT-0, -I, II, -III), MFGE8, TSG101, flotillin, PDCD6IP (ALIX), and tetraspanins molecules (CD9, CD63, CD81) have been used as biomarkers of exosomes [52, 53].

A notable feature of exosomal proteins is the maintenance of functional activities including antigen presentation, peptide, and protein cleavage [54]. Valadi *et al.* indicated that exosomes contain mRNAs and miRNAs, that transfer to recipient cells with the corresponding function for intercellular regulation [55]. Exosomal mRNAs can be translated and miRNAs can mediate RNA-silencing in target cells. Gene transfer and gene silence mediated by exosomes are specific to target cells that are found in one specific but not in another type of cell [55–57]. In addition, the relative abundance of proteins, mRNAs and miRNAs differs between exosomes and donor cells [58]. This implies active sorting into MVBs. For proteins, this can be achieved by mono-ubiquitination, localized in cholesterol-rich membrane micro-domains, or oligomerization of a higher order [47, 59]. Thus, exosomes constitute a most potent mode of intercellular communication that has become important for immunity, cell to cell spread of infectious agents and tumor progression [49, 54, 60, 61].

### Cargo and functional activities

Exosomes from different sources exhibit distinct variation in their cargo of proteins and nucleic acids. Malignant cancer cells releasing the exosomes contain tumor- specific proteins. For instance, exosomes from ovarian cancer patients’ ascites contain Her2/Neu and from melanoma secretion contain Mart1 [62]. Studies have shown that exosomes from the serum of ovarian cancer patients comprise 8 types of microRNAs (miR-21, miR-141, mir-200a, mir-200b, 200C, miR-203, mir-205, and miR-214). The level of microRNAs is similar in exosomes and the parent carcinoma cell, while it cannot be detected in health exosomes or cells. This investigation also indicates that exosomal miRNAs have diagnostic value for ovarian cancer [63]. In addition the diagnostic value of microRNAs has been found in other cancers [64] such as prostate cancer [65], esophageal squamous cell carcinoma [66] and malignant tumors in lungs [67].

The proteomic analysis demonstrates that package proteins of exosomes are specific and are completely different from cell apoptosis by the release of apoptotic bodies [68, 69]. The High Throughput Sequencing technology verifies that the miRNA is also subject to certain mechanisms rather than a random package of exosomes [70]. Many reports show exosomes involved in the regulation of a variety of physiological activities and pathological processes such as the immune system, tissue repair, nervous system of traffic [71], cardiovascular diseases, neuro-degenerative diseases as well as tumors [72]. These investigations have demonstrated that exosomes are active rather than passively produced by parent cells. They are the new messengers of intercellular communication.

## EXOSOMAL TETRASPANIN NETWORK

### Structure of tetraspanin

The transmembrane 4 super-family (TM4SF) or tetraspanins are small transmembrane proteins expressed in many species [73]. Tetraspanins are implicated in a diverse range of biologicalprocessesincludingphysiological cell adhesion, motility, activation and proliferation as well as pathological cancer metastasis and viral infection [74, 75]. The structure is postulated to cross the membrane 4 times during which there is a short N and C terminal tail; a small extracellular loop (ECL1) between transmembrane regions TM1 and TM2; a small intracellular loop (ICL) between TM2 and TM3; and a large extracellular loop (ECL2) between TM3 and TM4. The constant region of the ECL2 is responsible for dimerization of themselves, the variable region for associating non-tetraspanin molecular partners.

**Figure 1:**
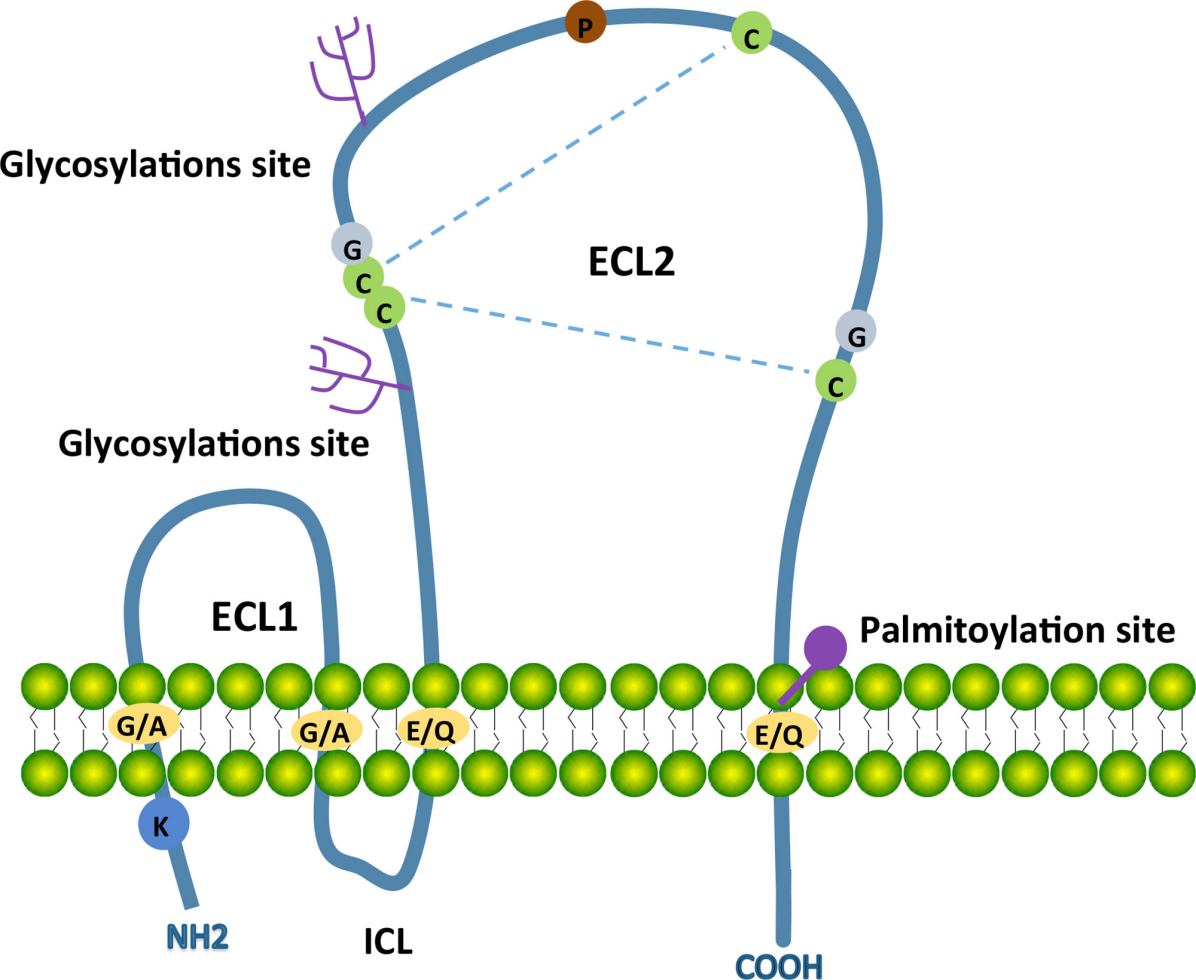
Schematic structure of most tetraspanins with key characteristics Tetraspanins contain four transmembrane domains (TM1 to TM4), a small extracellular loop (ECL1), a small intracellular loop (ICL) and a large extracellular loop (ECL2). The N-terminal and C-terminal tails are intracellular. Transmembrane domains contain conserved polar/charged residues (orange circles), ECL2 contains a highly conserved CCG motif and additional two conserved cysteine residues (green circles). Two disulphide bridges (blue dotted lines) can form between these cysteine residues for the folding of ECL2. ECL2 contains two glycosylations site (pink trees) andTM4 has a putative palmitoylation site (pink circle). The conserved residues including one proline (brown circle) and two glycine (grey circles) locate in ECL2 and one lycine locates in N-terminal tail.

Key features of tetraspanins include a highly conserved ‘CCG’ motif and an additional two or more cysteine residues in the ECL2 domain. TM regions contain polar residues to stabilize the tertiary structure [76]. Most tetraspanins are glycosylated in the region of ECL2, thought to impact tetraspanin function. Some tetraspanins may have palmitoylation cysteines in their transmembrane region. Palmitoylation is required for initiating tetraspanin-tetraspanin web formation [77]. Some tetraspanins contain a tyrosine-based sorting motif for intracellular compartment targeting that may lead to internalization via associated proteins [78] (Figure 1).

### Large protein complex of tetraspanins

Tetraspanins associate with themselves as well as with numerous transmembrane and/or cytosolic proteins to assemble into complexes [12]. Tetraspanin enriched membrane micro-domains (TEMs) comprising tetraspanin complexes act as platforms for signalling transduction [12, 79, 80]. The strength of molecular interaction in tetraspanin complexes can be grouped into three types: type I is direct protein-protein interaction with stronger association; type II is tetraspanin dimerization and tetraspanin or integrin interaction with mild association; type III is tetraspanin and several kinases interactions with weaker association and stabilizing by palmitoylation [76, 79]. Tetraspanins are associated with growth factor receptors, G-protein-coupled receptors (GPCRs), two members of immunoglobulin (Ig) super-family (EWI-F and EWI-2), and signal transduction molecules (PKC) [81, 82]. Integrins are the most prominent molecular partners of tetraspanins including α3β1, α4β1, and α6β1. Tetraspanins act as molecular facilitators to modulate the functions of associated molecules [83]. They influence cell invasiveness by modulating MMP production and cell adhesion through regulating biogenesis and traffic of corresponding molecules [84, 85]. Furthermore, several tetraspanins are also involved in the process of virus and parasite infections [86].

Exosomal tetraspanins engage in internalization, vesicle traffic and deviation from degradation in the proteasome [62, 87]. Clathrin and intersectin2 are required for Tspan8 TEM internalization [87]. The function of tetraspanins in cargo loading of exosomes has not been fully investigated. The recruitment of miRNA in Tspan8kd and CD151kd pancreatic cancer exosomes leads to minor differences. To address the question, the uniform exosomes need to be evaluated and scrutinized but not focusing on the single cell-type released exosomes. A few studies have concentrated on defining sub-populations of exosomes that facilitate exact targets [88].

In a cancer context, many studies indicate that Tspan8 promote cancer metastasis and angiogenesis [75, 89]. Furthermore, Tspan8 mediates tumor cell motility and invasion [72, 90]. Tetraspanin CD151 has been shown to be involved in tumor cell motility, invasion, metastasis and angiogenesis [69, 70]. CD9 is considered to be a tumor suppressor and facilitates tumor angiogenesis [76, 91]. Tetraspanin CD82 has been reported to be a tumor suppressor and a modulator of membrane heterogeneity [77, 92]. Some investigations support that CD63 is a cancer metastasis suppressor, while the functional role of CD63 needs more evaluation [79, 93] (Table 1).

**Table 1:** The functional roles of tetraspanin in cancer

Tetraspanin	Functional roles in cancer	Detect in exosomes	Key associated proteins	References
CD151	Tumour cell motility, invasion, metastasis, tumor initiation, promotion, progression, and angiogenesis	yes	Integrins α6β4, α6β1, α3β1	[35, 71, 72, 127]
Tspan8	Tumor growth, and angiogenesis, tumour cell motility, invasion, metastasis	yes	E-cadherin, claudin 7, EPCAM, α6β4 integrin, CD44v6 and EWIF	[35, 74, 77, 91, 92, 127]
CD9	Tumour metastasis suppressor, tumour angiogenesis, lymphangiogenesis and tumour growth	yes	EWI2, EWIF EPCAM, HB-EGF	[78, 89, 93]
CD82	Suppressor of tumour migration, invasion and metastasis	yes	Integrins α6β4, α4β1, α3β1	[79, 94]
CD63	Tumour metastasis suppressor	yes	Integrins α6β1, TIMP1, CD82, CD9	[81, 95]

## EXOSOME ASSEMBLY AND TETRASPANINS INTERNALIZATION

### Exosome assembly

In most mammalian cells, portions of the plasma membrane are regularly internalized into a membrane bound compartment as endosomes [94]. Parts of the membranes of some endosomes are subsequently internalized as smaller vesicles to become multi-vesicular bodies. The endosomes can become lysosomes or recycled back to the plasma membrane. Molecules from the plasma membrane can also become lysosomes for degradation or can be recycled back to the plasma membrane. Molecules are tagged with ubiquitin that can be recognized and sorted into lumenal vesicles by the ESCRTs [95–97]. Marked molecules in the lumen of endosomes will be degraded in lysosomes.

**Figure 2:**
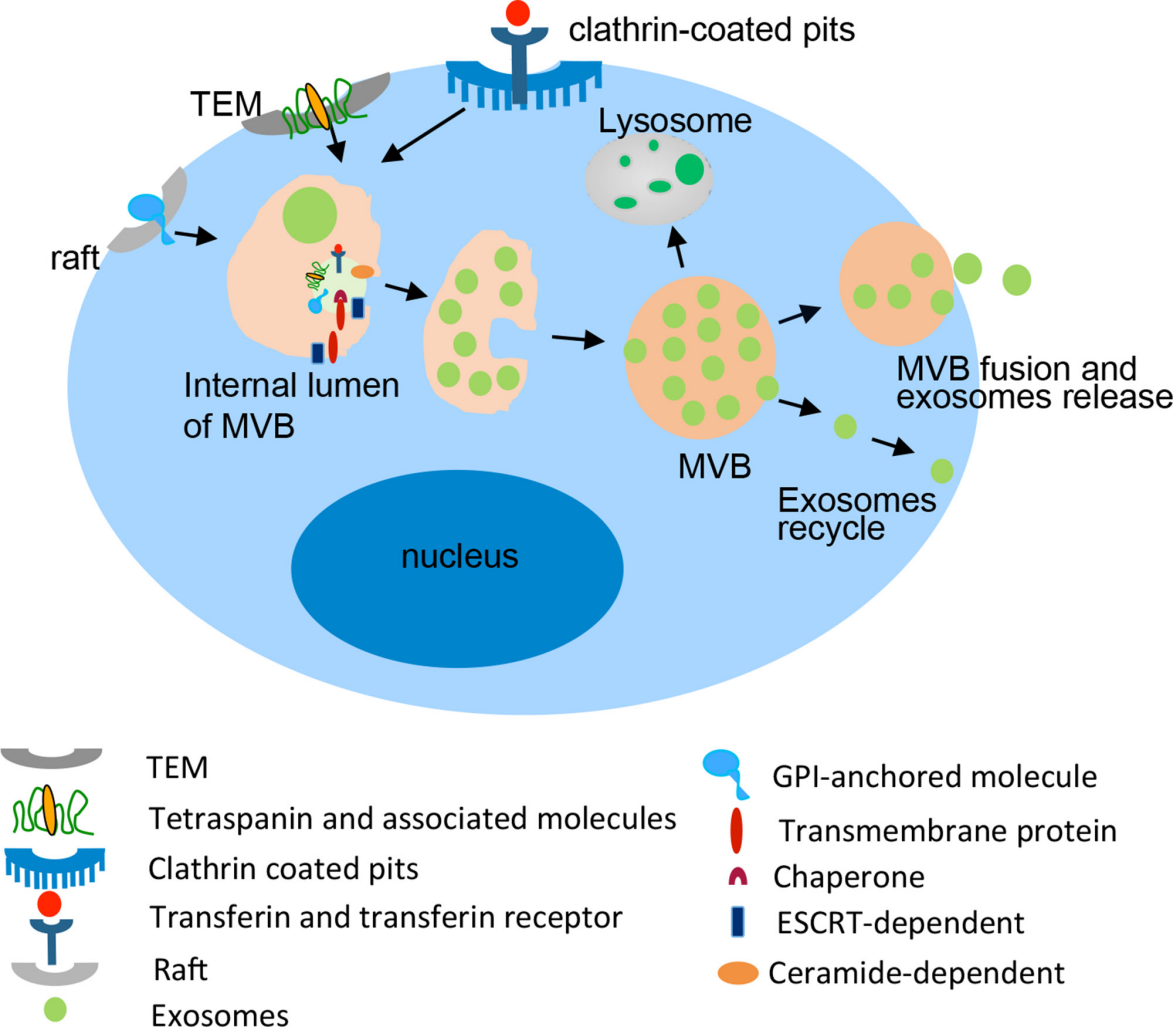
Biogenesis and composition of exosomes Exosomes derived from the intralumenal vesicles (ILVs) of MVBs, which formed by the late endosomes with the membrane proteins sorted by TEM, Lipid rafts, Clathrin coated pits and the endocytosis of cytoplasmic molecules dependent on ESCRT or ceramide. The membrane of MVBs fuses with the plasma membrane resulting in the release of exosomes into the extracellular environment.

The multi-vesicular body fuses with the cell memb-rane and releases the intralumenal endosomal vesicles into extracellular space that become exosomes [98]. Multi-vesicular bodies, internalized with endosomes membrane, comprise the lipid rafts micro-domain with GPI-anchored proteins [99]. Endocytosis, mediated by clathrin-coated pits, regulates the internalization of many molecules as transferin and transferin receptors as lumenal vesicles [100, 101]. Tetraspanins and tetraspanin-associated molecules locatedbyTEMs are sorted into small vesicles during the generation of MVBs. Cytoplasm protein can be internalized into MVBs via ESCRTs or ceramide-dependent pathways [54] (Figure 2).

### Internalization of tetraspanins into exosomes

Enrichment of tetraspanins in exosomes receives progressive attention in unraveling protein-protein and protein-lipid interactions, membrane dynamics and intracellular vesicle sorting [74, 78]. Besides the clustering potential ligands for signal transduction, tetraspanins may contribute to the assembly of exosomes. Tetraspanin internalization could influence the selectivity of exosomes assembly. For example, the content of proteins and mRNA is different in exosomes derived from the same cell line with or without transfection of Tspan8 cDNA [102]. Tetraspanins internalize into exosomes through specific molecules or motifs rather than through clathrin-coated pits and caveolae. A tyrosine-dependent internalization motif (YxxФ) or the c-terminal tail of tetraspanins and associated molecules are suggested to facilitate TEM-located tetraspanins internalization [78, 101, 103].

CD151 contains a sorting motif that allows rapid internalization. CD9 has no sorting motif whereas Tspan8 has one, but it is too closely located to bind the AP complex. In the resting cells, CD9 and Tspan8 are associated with CD151. The internalization of Tspan8 and CD9 is accompanied by CD151 during PMA stimulation [87]. Intersection-2 (INS2), a multi-modular protein, gets involved in clathrin-mediated endocytosis with Tspan8-associating α4 or α6β4 complex [104]. It is known that Intersections (INSs) contain Eps15 domains that interact with dynamin and synaptojanin to promote endocytosis [105]. Tspan8 associating with INS2, dynamin, α4, or β4 makes a major contribution to the generation of MVBs through the synthetic route and exosomes biogenesis [87, 102, 106].

## REGULATION OF TETRASPANINS IN EXOSOMES UPTAKE AND TARGET CELL REPROGRAMMING

### Exosomal tetraspanin-complexes contribute to target selection

Studies have shown that binding/uptake of exosomes in target cells is directed by specific mechanisms. It is likely that exosome uptake takes place in internalization-prone membrane micro-domains. New evidence indicates that the exosomal tetraspanin web is involved in the binding of target cell ligands and receptors. We have tested the uptake of 4 types of exosomes derived from pancreatic adenocarcinoma lines BSp73AS (AS) of a rat expressing different tetraspanins by target cells *in vitro* and *in vivo* (exosomes from AS, AS-Tspan8, AS-Tspan8/CD9n, AS-Tspan8/Integrinβ4) [40]. The results demonstrated that the minor differences in the composition of exosomes cause the distinct uptake by target cells. AS-Tspan8 exosomes preferentially bind to rat endothelial cell, while the plus β4 bind to stromal cell and AS exosomes bind to fibroblasts *in vitro*. The *in vivo* uptake of the selectivity of target cell is more surprisingly significant with differences between the 4 exosomes types [40, 107]. Pull-down experiments unraveled the membrane molecules involved in exosomes binding as MFGE8, CD44, CD49e, CD54, CD56, and CD106. In addition, phosphatidylserine (PS) was exposed on the outer membrane of exosomes support uptake [107]. Similar fusion/fission machineries are common for internalization of exosomes in parent cells and uptake of exosomes by recipient cells [47]. The lipid ligands and proteins of exosomes bind with cell surface receptors by activating the signal to deliver the factors to target cells [108].

**Figure 3:**
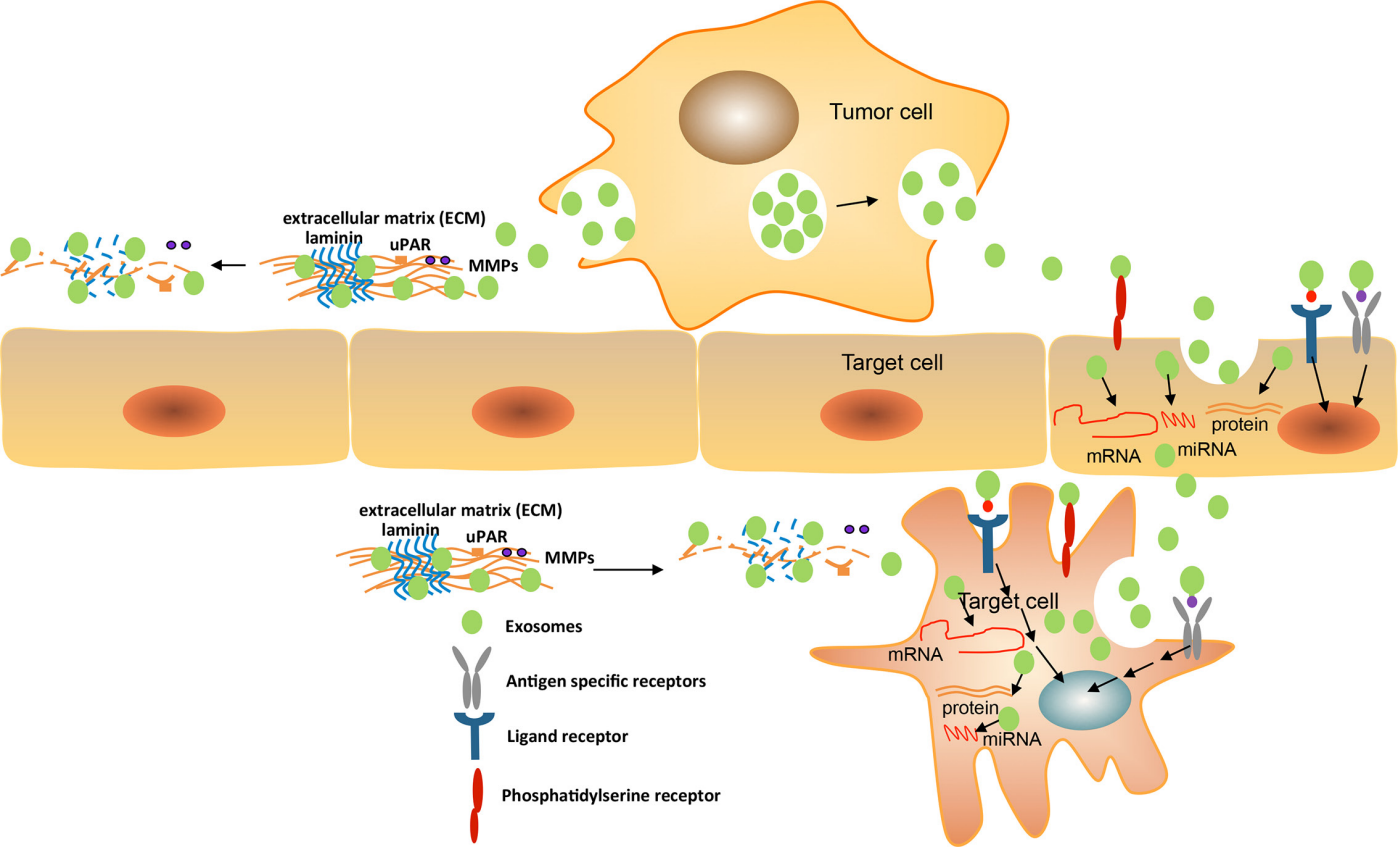
Exosomes mediate target cell reprogramming and alter cancer microenvironment Exosomes can uptake into the target cells (endothelia cell or cancer stroma cell) by the membrane fusion mechanisms, the interaction of phosphatidyl serine and its receptor, the binding of ligands and receptors, and the transfer of both the specific molecules and antigens. Exosomes can directly bind cell surface receptors by interaction with exosomal ligands that may activate the cascade of signaling transduction, thus initiates the expression of specific genes by transcription factors regulation, oncogenes activity, and the immune regulation. Exosomes containing various molecules including proteins, antigen and cytokines can be delivered to recipient cells during internalization that allow regulating target cells. Exosomal mRNAs and miRNAs are functional and can express into new proteins or regulate specific gene expression in recipient cells. The exosomal components may mediate target cell reprogramming. In addition, exosomes enriched complex of tetraspanin/proteinase including MMPs, uPAR, TACE, and ADAM17 with full activity regulate cell motility and invasiveness via extracellular matrix degradation. The matrix proteins as laminins, collagens may be degraded by proteinase to create a path for cell migration or invasion.

Exosomes also can be involved in immunity regulation by the presentation of antigens or the transfer of antigens and major histocompatibility complex (MHC) molecules [52, 109]. The entrance of exosomes is dependent on transmembrane proteins including the interaction of ligands and receptors, the membrane fusion and the endocytosis process [110]. The subsequent mechanism of exosomes uptake is still poorly understood. Our studies demonstrate that the tetraspanin-integrin complex contributes to the selectivity of exosomes binding to target cells [40, 102]. David Lyden *et al.* revealed the exosomal integrins that are involved in target cell selection. Exosomal integrins activate the signalling pathway of Src phosphorylation and pro-inflammatory S100 gene expression. Integrins α6β4 and α6β1 are responsible for lung metastasis while integrin αvβ5 is linked to liver metastasis (Figure 3) [111].

### Exosomes uptake promote target cell reprogramming

Exosomes containing selected patterns of mRNA and miRNA imply that a dynamic regulation of gene expression can occur in the recipient cell. Besides the mRNA, a subset of miRNA can be transferred by exosomes derived from mouse embryonic stem cells to mouse embryonic fibroblasts *in vitro* [112]. Investigations suggested that transferring of miRNA mediated by exosomes may alter the expression of gene products in target cells. Cancer-associated fibroblasts (CAFs) exosomal miRNAs (miRNAs -21, -378e, and -143) can be delivered to breast cancer cells increasing the capacity of mammospheres formation, the expression of biomarker of stem cell and epithelial-mesenchymal transition (EMT) and ability of anchorage-independent cell growth [113]. The transfer of genetic information by exosomes play a pivotal role for reprogramming of target cells.

Aside from transfer miRNA, the exosomal content can be delivered into the target cell leading cell reprogramming. Zhou et.al demonstrated that exosomes derived from human embryonic stem cells can reprogram in malignant cancer cells. The cargo of exosomes are able to be transfered into target cancer cells and induce the expression of SOX2, OCT4 and Nanog. They also indicate that human embryonic stem cell-derived exosomes played a dose-dependent decrease of cancer cell growth and tumorigenicity [114]. In tumor biology, exosomes can be regarded as signalosomes by stimulating tumor growth, angiogenesis, matrix remodeling, immune escape and metastasis. Exosomes can induce the expansion of regulatory T cells or apoptosis of CD8^+^ T cells to initiate tumor cell immune escape [115, 116].

## CANCER MICRO-ENVIRONMENT ALTERATION MEDIATING METASTASIS

### Exosomes mediate communication of stromal and cancer cells

Cancer micro-environment is composed various cells including cancer associated fibroblast (CAFs), endothelial cells, tumor associated macrophages (TAMs), as well as extracellular matrix and other factor. Cancer metastasis is mediated by interactions among cancer cells and cancer micro-environment. Exosomes can regulate distant cellular communication, which modulate the cross-talking between the resident cell via various oncogenic signaling pathways.

Cancer cells derived exosomes transfer TGF-β into CAFs to develop myofibroblastic phenotype, which can support cancer growth. The myofibroblastic phenotype of CAFs in cancer micro-environment is TGF-β-SMAD signaling dependent. It is also reported that phosphatidylserine mediated fusion of exosomes and endothelial cell facilitate the expression of EGFR on cancer associated endothelial cells. Also, TAMs secret the VEGF into cancer microenvironment to regulate angiogenesis. Exosomes play a pivotal role in maintains a balance between pro- and anti-angiogenic factors the cancer microenvironment [102].

### Exosomes remodeling host extracellular matrix

Exosome binding can alter cancer micro-environment by modulating the host extracellular matrix (ECM). Their abundant enzymes affect the host matrix by degrading proteins [117]. Exosomes from a highly metastatic rat pancreatic cancer line (ASML cells) have recovered high levels of uPAR, MMP2, MMP3, MMP9, MMP14, and ADAM17 proteinases. *In vitro* experiments demonstrate that ASML^wt^ (ASML wild type cells without any molecular modifications) exosomal proteases are functional degradation for the ECM of stromal lines and endothelial cells, and for several proteins as collagen, fibronectin and laminins [32, 33]. Furthermore, exosomal proteases may contribute to matrix protein maturation such as the ADAMTS family for collagen maturation [118]. Also, they are possibly involved in generating fragments of matrix proteins to exert distinct functions such as laminin fragment promoting cell motility and small HA fragments promoting an inflammatory milieu [119, 120]. Taken together, exosomal proteases are critical elements for cross talk between tumors and the host matrix by creating space for cell motility and cancer metastasis (Figure 3).

Cancer cell line AS, expressing Tspan8-derived exo-somes, promotes endothelial cell proliferation and cable formation. Co-culture of endothelial cell and AS-Tspan8 exosomes induces migration-related expression of molecules as CCR1, CCL20, CXCL5, and MIF [102]. MIF may stabilize HIF1α to promote the expression of angiogenic growth factor [121]. Similar angiogenic effects were observed with exosomes derived from different tumor lines expressing Tspan8 and integrin α4. This indicated that the exosomal Tspan8 integrin α4 complex was a decisive factor in exosomes-induced angiogenesis without the defined angiogenic factors. Angiogenesis induction is independent of the release of angiogenic factors but essentially depends on Tspan8-containing exosomes. As consistent components of exosomes, tetraspanins present a clue for the exosomal angiogenic function.

Animal experiments demonstrated that the highly metastatic line ASML has a slow local and metastatic growth in lymph nodes and lungs. A CD44 variant isoform selective knockdown of this line (ASML-CD44v ^kd^) loses the capacity of metastasis formation [122]. The exosomes secreted by the knockdown line do not exert the fully metastatic phenotype while their co-cultures facilitate knockdown cells settling in lymph nodes and lungs. ASML cell-derived exosomes include enriched tetraspanins, integrin α6β4, EpCAM, claudin-7, c-Met, uPAR, MMPs, and other molecules. Results indicated the association of integrin α6β4, EpCAM and claudin-7 with Tspan8 in ASML exosomes. ASML exosomes up-regulate several adhesion molecules including integrin α4, CD44v6, chemokines, growth factors, angiogenic factors and proteases as well as preferentially targeted lungs and lymph node stromal cells [123, 124]. The double knockdown of ASML (ASML^dk^ -Tsp8-CD151) by Tspan8 and CD151 lacks the capacity of metastasis [125]. ASML exosomes can recover the metastatic capacity of ASML^dk^-Tsp8-CD151 and ASML-CD44v^kd^ cell [33]. These studies demonstrated how ASML-derived exosomes support premetastatic niche formation. Tetraspanins are involved in the preparation of premetastatic niche formation.

## CONCLUSION

Exosomes are small vesicles with large functions and play a pivotal role in inter as well as intracellular communications. Though the complex role of exosomes in cancer biology is slowly being established, knowledge on their mode of interaction with the cancer micro-environment is still limited. It is well known that exosomes recruit diverse bioactive molecules including proteins, mRNA and miRNA of donor cells. However, little is known as to how exosomes influence distant cells or alter the cancer micro-environment. The enrichment of tetraspanins in exosomes may provide a clue to uncover the mechanism of their selective uptake by target cells. The central feature of tetraspanins is a molecular facilitator that associates with other tetraspanins, integrins and additional transmembrane proteins in TEM. Also, it facilitates the recruitment of signal molecules. TEM assembly in exosomes surface and internalize tetraspanins is through the process of endocytosis. Exosomal tetraspanins direct the selection of target cells and the preparation of pre-metastatic niche formation. The complex content of exosomes exerts the reprogramming of target cells by translating new protein and regulating new gene expression.

The nano-scale particle of exosomes is extensively beneficial to apply to the process of diagnosis and therapeutics. Tumor-derived exosomes in all body fluids may provide an alternative for a reliable diagnostic tool. Cancer-specific exosomal protein and miRNA have received much attention for being possible diagnosis markers. The potential of delivering therapeutic content and biological membranes without invoking an immune response drives exosomes to be the new cargo for therapeutics. As the most important intercellular communicators, exosomes modulate, instruct, and reprogramme their surroundings as well as distant organs. The tailored exosomes may offer a potent therapeutic delivery system. In the last several years, research on exosomes have attracted an ever-increasing and overwhelming interest. The knowledge of exosomes will open new doors in the field of cancer biology including diagnosis and therapy.
